# Fluid dynamics of droplet generation from corneal tear film during non-contact tonometry in the context of pathogen transmission

**DOI:** 10.1063/5.0061956

**Published:** 2021-09-14

**Authors:** Durbar Roy, Sophia M, Abdur Rasheed, Prasenjit Kabi, Abhijit Sinha Roy, Rohit Shetty, Saptarshi Basu

**Affiliations:** 1 Department of Mechanical Engineering, Indian Institute of Science, Bengaluru, KA 560012, India; 2 Interdisciplinary Centre for Energy Research, Indian Institute of Science, Bengaluru, KA 560012, India; 3 Imaging, Biomechanics and Mathematical Modelling Solutions lab, Narayana Nethralaya Foundation, Bengaluru, KA 560010, India; 4 Cornea and Refractive Surgery Services, Narayana Nethralaya, Bengaluru, KA 560010, India

## Abstract

Noninvasive ocular diagnostics demonstrate a propensity for droplet generation and present a potential pathway of distribution for pathogens such as the severe acute respiratory syndrome coronavirus 2. High-speed images of the eye subjected to air puff tonometry (glaucoma detection) reveal three-dimensional, spatiotemporal interaction between the puff and tear film. The interaction finally leads to the rupture and breakup of the tear film culminating into sub-millimeter sized droplet projectiles traveling at speeds of 0.2 m/s. The calculated droplet spread radius (∼0.5 m) confirms the likelihood of the procedure to generate droplets that may disperse in air as well as splash on instruments, raising the potential of infection. We provide a detailed physical exposition of the entire procedure using high fidelity experiments and theoretical modeling. We conclude that air puff induced corneal deformation and subsequent capillary waves lead to flow instabilities (Rayleigh–Taylor, Rayleigh–Plateau) that lead to tear film ejection, expansion, stretching, and subsequent droplet formation.

## I. INTRODUCTION

The impact of gas jets on the surface of deep liquid pools is well studied.^1–4^ However, the impact of gas jets or unsteady puffs on thin films is relatively unknown. Generally, jets are composed of vortex rings and their effect on solid walls has been pursued by scientists in the past.^5,6^ Such interactions occur when drying painted walls with an air gun or more commonly when the wind hits the tear film on the eye. Another common example is the splashing of tears during the use of air puff tonometry. Tonometers are devices used by ophthalmologists to diagnose the early onset of glaucoma based on the measurement of the intra-ocular pressure (IOP).^7,8^ The early models of the tonometer were contact based but along with progressive improvements to accuracy,^9^ many modern tonometers feature a noninvasive method of diagnosis, using air to applanate the eye. Air puff tonometers are devices that generate a short puff and measure corneal applanation to gauge the IOP. A clinical study by Britt *et al.*^10^ demonstrated tears splashing from a human subject during air puff tonometry. Since viruses like human T-cell leukemia/lymphotropic virus type III (HTLV-III)^11^ and severe acute respiratory syndrome (SARS)-coronavirus (CoV)-2^12,13^ can be present in human tears, it was conjectured that such an event is a possible source of infection. Shetty *et al.*^14^ showed using clinical studies that droplets are visibly ejected in the case of watery eyes but not for dry eyes. However, the clinical studies do not attempt a physical analysis of the underlying fluid dynamics involved in the tear film breakup and tear ejection. The experimental complexity of imaging the spatiotemporal scales involved [$O(10^{-3})\;s,\;O(10^{-4})\;m$] without posing a serious threat to the human eye limits the size of droplets observed. However, it would be incorrect to conclude that dry eyes do not lead to tear ejection. This motivates us to rigorously analyze the complex, multiphase phenomenon from first principles.

The ongoing pandemic is a secondary source of motivation for this work. Since December 2019, scientists have joined clinicians in exploring every aspect of the SARS-CoV-2. The virus SARS-CoV-2 is transmitted between humans through nasal and oral discharges, in the form of droplets.^15–17^ Such droplets are routinely expelled by human beings during coughing, talking, breathing, and sneezing. Scharfman *et al.*^18^ showed that the breakup of droplets happens outside the respiratory tracts in a violent exhalation processes. They showed various mechanisms of droplet breakup such as sheets, bags, and ligament dissociation. Bourouiba *et al.*^19^ also studied the fluid dynamics of violent expiratory events using high-speed light scattering technique and found the flow to be highly turbulent and multiphase. Further, a theoretical analysis of pathogen bearing droplets interacting with the turbulent puff was conducted by Chaudhuri *et al.*^20^ who developed a first principles model to explain the relationship between respiratory droplets and pandemics. The general consensus is that smaller droplets can be directly inhaled while larger droplets settle to form fomites.^21,22^ Recent studies by Sadhu *et al.*^23^ demonstrate SARS-CoV-2 virus in human tears, although evidence to the contrary has been presented by Sun *et al.*^24^ This further necessitates a focused, rigorous study of how tonometry can create micro-droplets.

**FIG. 1.**
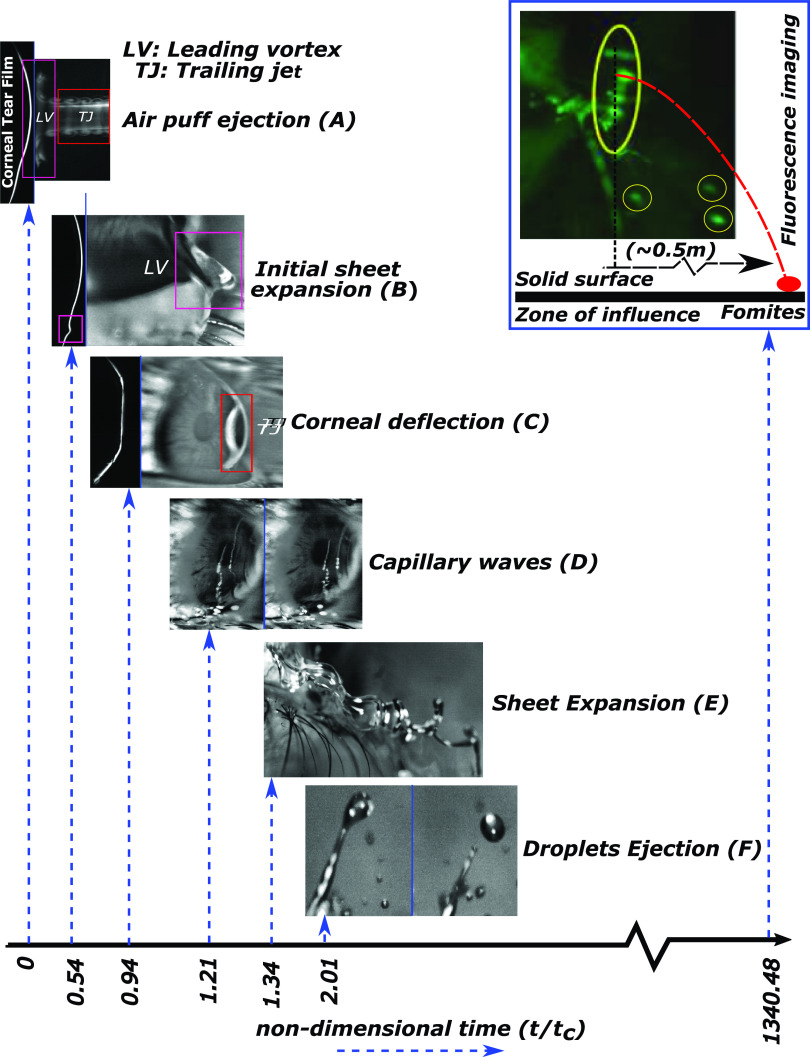
Different phases during non-contact tonometry. Capillary time scale $t_{c}=\rho_{l}^{1/2}R^{3/2}\sigma_{al}{}^{-1/2}$ has been used to normalize time. Phase A represents the air puff generation from the nozzle and its propagation. Phase B consists of the initial sheet expansion due to the approaching leading vortex. Phase C represents the central corneal deflection due to the trailing jet. Phase D shows the ensuing propagation of capillary waves on the corneal surface. Phase E shows the subsequent sheet expansion, and phase F shows the droplet ejection regime due to Rayleigh–Plateau instability. The droplets generated travel under the influence of its initial linear momentum, gravity, and air resistance, and can propagate a maximum distance of 0.5 m. Long exposure fluorescence imaging was also used to visualize the droplets formed. Multimedia view: https://doi.org/10.1063/5.0061956.1
10.1063/5.0061956.1

**FIG. 2.**
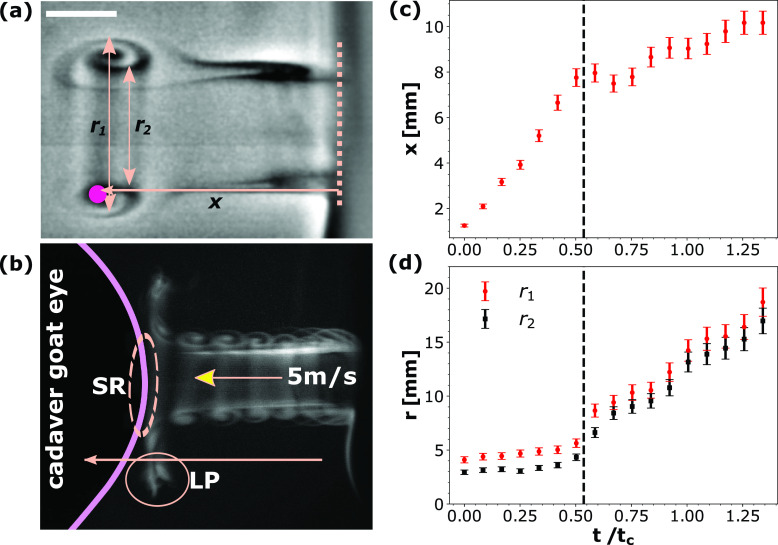
Air puff characterization using cadaver goat eye. (a) Smoke flow visualization of the leading vortex and the trailing jet. The position of the vortex is denoted as *x* while $r_{1}$ and r2 indicate its size. Scale bar equals 3 mm. (b) Superimposed image sequence illustrates the vortex interaction with the eye. LP denotes the low pressure region created due to the approaching leading vortex and SR denotes the stagnation region. (c) Plot of vortex position with time. (d) Plot of radial expansion ($r_{1}$ and r2) of the leading vortex with time. Capillary time scale $t_{c}=\rho_{l}^{1/2}R^{3/2}\sigma_{al}{}^{-1/2}$ has been used to normalize time.

We have used high-speed visualization to image the sequence of events leading to the tear film ejection in healthy human subjects. Commercially available eye drops (in consultation with ophthalmologists) were administered to the subjects prior to each puff. Excess amount of artificial tear accumulates near the lower eye lid (due to gravity), forming a thick film (0.5–1 mm). Control experiments with dry eyes were also performed as shown in Fig. 1. We have used capillary time scale $t_{c}=\rho_{l}^{1/2}R^{3/2}\sigma_{al}{}^{-1/2}$ to normalize time, where $R∼O(10^{-3})\;m$ is the characteristic length of the cornea, $\rho_{l}$ is the density of tear film, and $\sigma_{al}$ is the air–liquid surface tension. For water, the capillary time scale is $t_{c}∼3.73\;\text{ms}$. The puff from the tonometer is visualized as a thin vortex with a trailing jet [see Fig. 2(a)]. On impingement, it causes the tear film in the lower eye lid to expand and eject outward. The ejected tear sheet continues to expand and breaks up into droplets of various sizes and velocities. Due to the effect of its initial linear momentum, the droplets travel in various directions under the combined action of gravity and environmental draft. We decimate the event into several stages as described in Sec. II. Our primary objective focuses on the fluid dynamical mechanisms that generate droplets of various shapes and sizes during air puff tonometry. The maximum spread radius of the droplets ejected during tonometry can be predicted using the size and velocity distribution of the droplets generated. The droplets' size and velocity distributions generated during non-contact tonometry are reported, which may help the ophthalmologist and eye care practitioners follow specific safety protocols while undertaking these procedures to prevent pathogen transmission. Droplets in the size range of approximately 0.2–3 mm, traveling at a speed of 0.2 m/s are generated during tonometry of watery eye. Such droplets can have a maximum spread range of 0.5 m under normal operating procedures.

## II. RESULTS

The various phases of the tear sheet ejection and breakup are shown in Fig. 1 along with associated multimedia view (refer multimedia view Fig. 1 here) for a complete overview. During phase A, the leading vortex from the nozzle reaches the eye at a distance of 11 mm in 2.6 ms traveling at an average speed of 5 m/s. This creates a transient reduction in the air pressure near the eye, causing the accumulated tear film to expand for about 3 ms (phase B) before the trailing jet from the puff impinges on the cornea. The trailing jet impingement causes corneal deformation (phase C)^25,26^ that lasts for 7.5 ms and initiates phase D, by creating capillary waves on the corneal surface. These waves travel outward in concentric circles^27^ for about 3 ms. In the next phase E, the initial tear sheet, reinforced by the traveling capillary waves, expands and undergoes bag breakup^28,29^ as well as Rayleigh–Taylor instability to form expanding ligaments. This phase lasts for approximately 5 ms. The last phase F consists of finger like structures (from Rayleigh–Taylor instability) of the sheet disintegrating into droplets due to Rayleigh–Plateau breakup.^30–33^ The time scale of this phase is approximately 3.5 ms. The size and velocity distributions of the daughter droplets are tracked (for repeatability) till they disappear from the field of view. Droplets in the size range of 0.2–3 mm traveling with an average velocity of 0.2 m/s were detected. Such droplets may carry pathogens and cause infection. Now, we discuss all the phases separately in Secs. III A–III F.

## III. DISCUSSION

### A. Vortex ring trajectory toward the eye (phase A)

Smoke from a burning incense was used to both visualize as well as track the air puff issued from the tonometer. A cadaver goat eye replaced the human subject (see methods for details). Figure 2(a) shows the leading vortex and the trailing jet, outlined in the smoke screen. The propagating vortex was tracked as a function of time by plotting the horizontal position *x* of the vortex core [Fig. 2(a); pink dot], referenced from the exit plane of the tonometer nozzle [Fig. 2(a); dashed vertical line]. The outer and the inner diameters of the leading vortex [Fig. 2(a); *r*_1_ and *r*_2_, respectively] were also tracked to observe the vortex expansion as it moves away from the exit plane. Figure 2(b) shows the interaction between the vortex and the cornea center, which forms the stagnation region (SR) and the low pressure (LP) regions near the upper and lower eye lids due to the shearing effect of the vortex. Vortex–cornea interaction causes the average velocity of propagation of the vortex to reduce from ∼ 5 to ∼ 0.5 m/s as shown in Fig. 2(c). The vortex also expands (*r*_1_ and *r*_2_) upon reaching the cornea as shown in Fig. 2(d).

### B. Initial sheet ejection (phase B)

Tears protect the eye against small scale foreign invasion. They are composed of poly-electrolytes, mucus and lipids.^34,35^ When secreted in normal amount (dry eye in this present context), they form a thin film of $L_{\mathrm{dry}}∼$ 5–30 *μ*m.^36–38^ The adhesive force between the tear film and the cornea is strong.^39^ We evaluate the stability of the tear film by calculating the Bond number, which scales the effect of gravity against the capillary forces. The Bond number in the case of dry eye is approximately $Bo_{\mathrm{dry}}=\Delta \rho gL_{\mathrm{dry}}^{2}/\sigma_{al}∼10^{-4}$. We have used the acceleration due to gravity, $g=10\;{m/s}^{2}$; difference between the density of the tear $\rho_{l}$ and the surrounding air $\rho_{a}$ as $\Delta \rho =\rho_{l}-\rho_{a}∼O(10^{3})\;{\text{kg}/m}^{3}$; and the liquid–air surface tension $\sigma_{al}∼O(10^{-2})\;N\;m^{-1}$. A low Bond number indicates that gravity does not affect the vertical tear film under dry eye conditions. In the case of a watery eye, the tear film ($L_{\mathrm{watery}}=0.5-1\;\text{mm}$) has a higher thickness at the bottom. The corresponding Bond number for watery eye is approximately $Bo_{\mathrm{watery}}=\Delta \rho gL_{\mathrm{watery}}^{2}/\sigma_{al}∼10^{-2}$. When the leading vortex reduces the outer pressure field, it favors ejection of the tear film as observed in phase B. The complete analysis of the sheet expansion along with the initial sheet ejection is presented later in phase E.

### C. Corneal deflection (phase C)

**FIG. 3.**
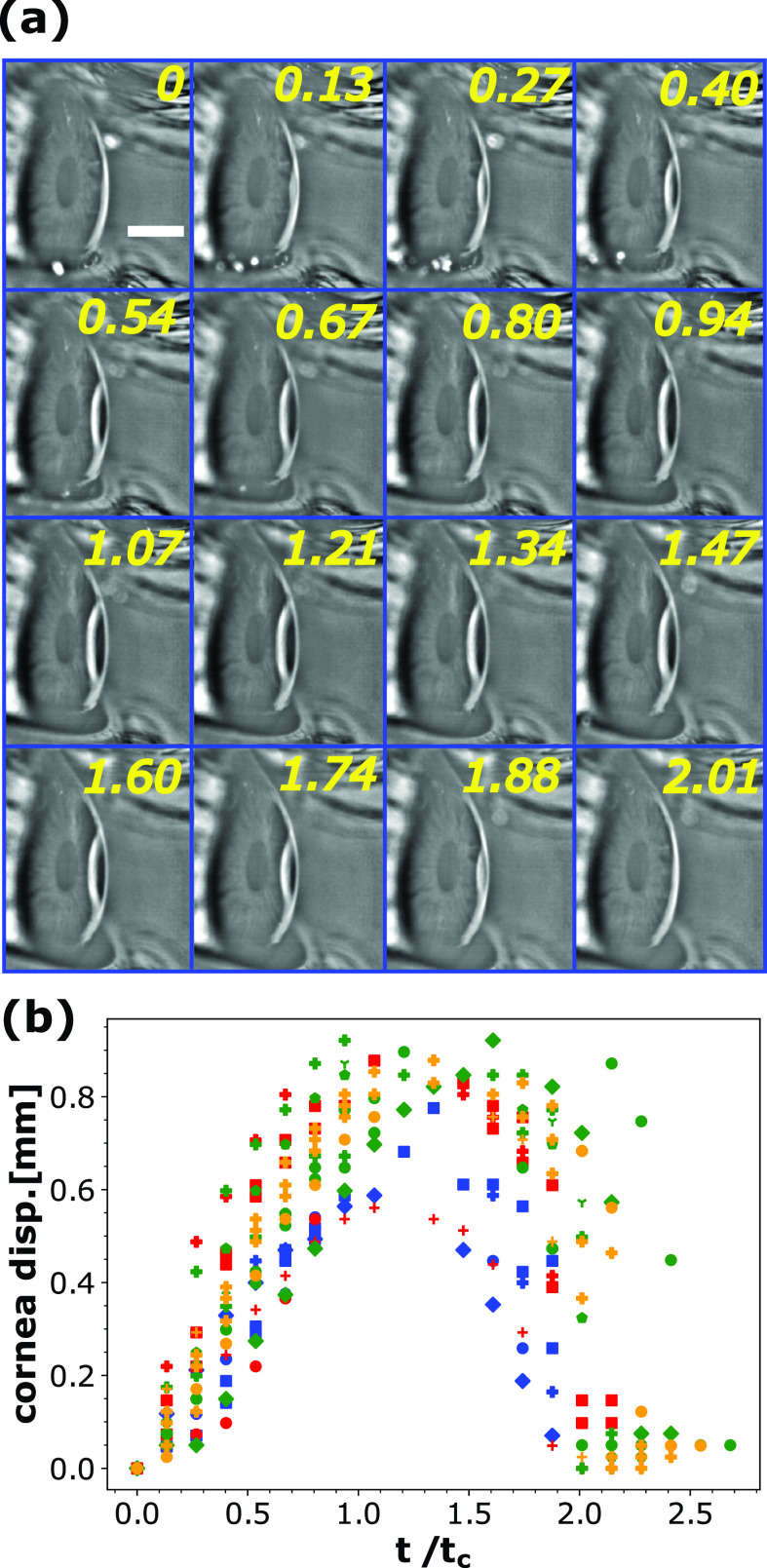
Corneal deflection by jet impingement. Capillary time scale $t_{c}=\rho_{l}^{1/2}R^{3/2}\sigma_{al}{}^{-1/2}$ has been used to normalize time. (a) Magnified, high-speed snapshots of complete deflection process. Time stamps are non-dimensional. Scale bar equals 3 mm. (b) Corneal deflection is plotted with time. The different colors (red, green, blue, and yellow) pertain to four different human subjects.

The vortex is followed by the jet, whose kinetic energy per unit volume is converted into the stagnation pressure near the SR [Fig. 2(b)], causing an elastic deformation of the cornea as shown in Fig. 3(a). Figure 3(b) shows the central corneal deflection profile as a function of time. The curve peaks at 4 ms and the entire deformation sequence spans 7.5 ms. The maximum corneal deflection varies between 0.6 and 0.9 mm [Fig. 3(b)] depending on the IOP of the subject and shows considerable variation^40^ not considered in this work. The precise value of central corneal deflection depends on various bio-mechanical and biological parameters like IOP, corneal hysteresis, and the anatomy of the cornea to name a few. Previous studies have shown that the central corneal thickness can affect IOP, which as a result can change the central corneal deflection. The average value of the central corneal thickness is of the order of 0.534 mm.^41^ Various chronic diseases can have effect on the central corneal thickness also. The corneal thickness has a linear dependence with IOP with a positive correlation.^42–44^ While several models of corneal deflection are available,^25,26^ we are concerned with the qualitative role played by the corneal deflection in creating the capillary waves as discussed in Sec. III D.

### D. Capillary waves (phase D)

The central corneal deflection is accompanied by the formation of surface capillary waves due to the impact pressure of the impinging jet and the restoring action of the surface tension of the tear film. The waves travel along the corneal surface in an axisymmetric manner about the center of the impact zone. The symmetry exists locally on a small spatiotemporal scale relative to a coordinate system centered about the impact region. Hence, a one-dimensional model is a good approximation to decipher the relevant velocity scales of the expanding ripples. The dispersion relations for pure capillary waves (1D relation) is given by^45^

$$\omega_{c}=\sqrt{\frac{\sigma_{al}}{\rho_{a}+\rho_{l}}}k_{c}^{3/2},$$ (1)

where $k_{c}=2\pi /\lambda_{c}$ is the wavenumber, which is inversely proportional to the wavelength ($\lambda_{c}$) of the capillary waves. Equation (1) implies that smaller wavelengths have higher angular frequency compared to larger ones. The speed with which capillary waves of a given wavenumber/wavelength propagate is known as the phase velocity and expressed as

$$v_{\mathrm{phase}}=\frac{\omega_{c}}{k_{c}}=\sqrt{\frac{\sigma_{al}}{\rho_{a}+\rho_{l}}}k_{c}^{1/2}$$ (2)

while the group velocity is

$$v_{\mathrm{group}}=\frac{d\omega_{c}}{dk_{c}}=\frac{3}{2}\sqrt{\frac{\sigma_{al}}{\rho_{a}+\rho_{l}}}k_{c}^{1/2}.$$ (3)

**FIG. 4.**
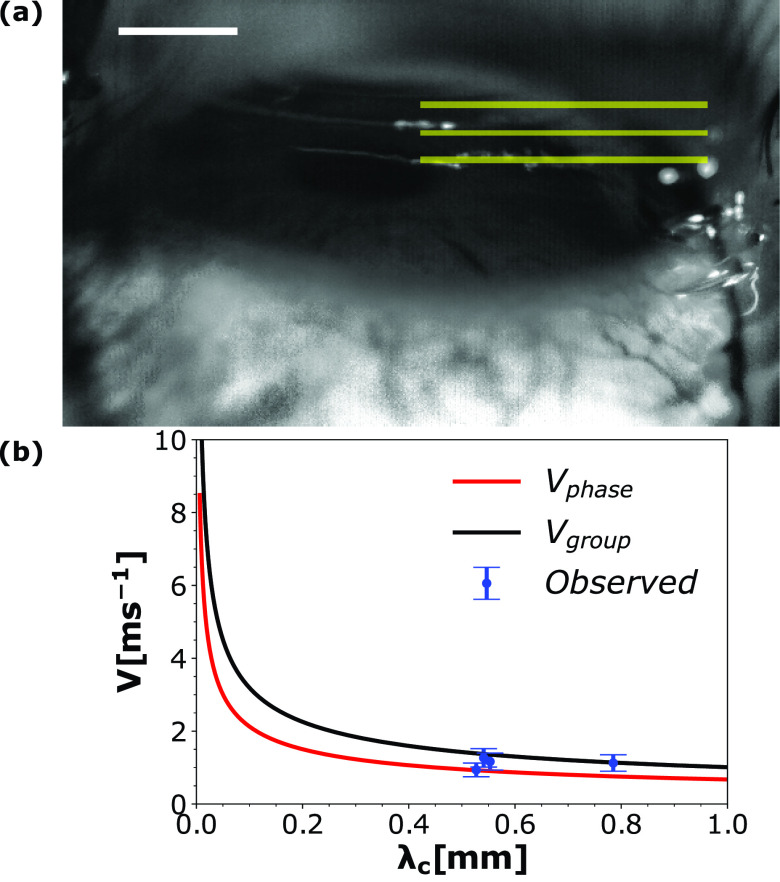
(a) The distance between consecutive horizontal dashed lines gives a measure of the wavelength $\left(\lambda_{c}\right)$ associated with capillary waves. Scale bar represents 3 mm. (b) Plot of theoretical phase velocity ($V_{\mathrm{phase}}$) and group velocity ($V_{\mathrm{group}}$) for capillary waves as a function of wavelength and corroborated by observed propagation rates (×) at discrete wavelengths.

**FIG. 5.**
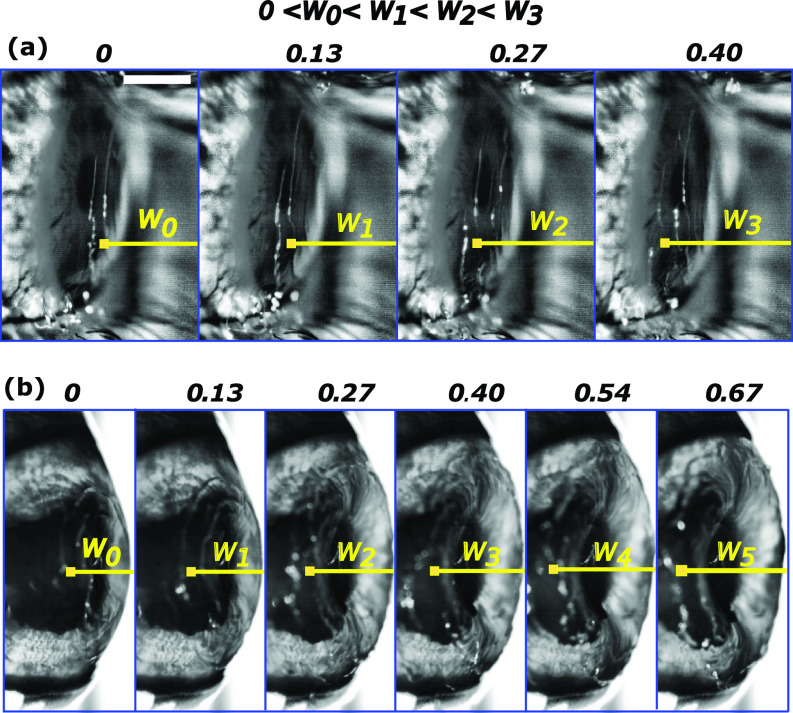
Image sequence of capillary wave propagation after jet impingement on corneas with different values of IOP. (a) Shows the waves on a human eye with a mean IOP of 15 mm of Hg. (b) Capillary waves on a cadaver goat eye with a mean IOP of 5. All time stamps are non-dimensional. Capillary time scale $t_{c}=\rho_{l}^{1/2}R^{3/2}\sigma_{al}{}^{-1/2}$ has been used to normalize time. The position of the crest (*w*_0_, *w*_1_, *w*_2_, *w*_3_), at different times, with respect to a fixed reference is used to calculate the wave velocity. Scale bar equals 3 mm.

The wavelength associated with the capillary waves $\left(\lambda_{c}\right)$ was calculated from various close up images as shown in Fig. 4(a). The distance between consecutive horizontal dashed yellow lines (distance between consecutive crests of capillary waves) gives a measure of $\lambda_{c}$. Equations (2) and (3) are numerically plotted in Fig. 4(b), which shows that smaller wavelengths have higher propagation rates. Figure 5(a) shows the time series of the propagating capillary waves on the human eye. The capillary wave peak was tracked from a fixed reference datum. *w*_0_, *w*_1_, *w*_2,_ and *w*_3_ represent the location of the same crest on the capillary wave 0.5 ms apart. Forward difference was used to calculate the average velocity of the wave. The experimentally determined wave velocity shows good match with the theoretical values [*O*(1) m/s] shown in Fig. 4(b). As discussed in Sec. III B [initial sheet ejection (phase B)], the Bond number in both the dry and watery eye is of the order of $10^{-4}$ and $10^{-2}$ due to very thin tear film thickness, suggesting that surface tension is at least two orders of magnitude stronger than gravity. Therefore, surface tension is the only force that acts to stabilize the tear film against any disturbance caused by external air pressure field. Putting things in a more general framework including both gravity and surface tension, the wavelengths of the capillary waves detected were in the range of 0.5–0.8 mm, which is less than a quarter of the critical wavelength of water waves given by $\lambda_{m}=2\pi {\left(\sigma /\rho g\right)}^{1/2}$. The critical wavelength for water is $\lambda_{m}=17\;\text{mm}$. The wavelengths lying below a quarter of $\lambda_{m}$ falls in the category of capillary waves. A very good discussion is provided in waves in fluids by Lighthill and Lighthill.^46^ It could be conjectured that the variation in the corneal deflection, as shown in Fig. 3(b), should affect the wave characteristics. This is disproved using similar experiments with the cadaver goat eye. Figure 5(b) shows that the corneal deflection is higher in a cadaver goat eye due to its inherently lower IOP (3–7 mm Hg) when compared to human subjects (12–22 mm of Hg). However, the experimental wave velocity is found to be of the same magnitude as those observed in human eye.

### E. Expansion of sheet leading to rim instability (phase E)

#### 1. Sheet expansion

**FIG. 6.**
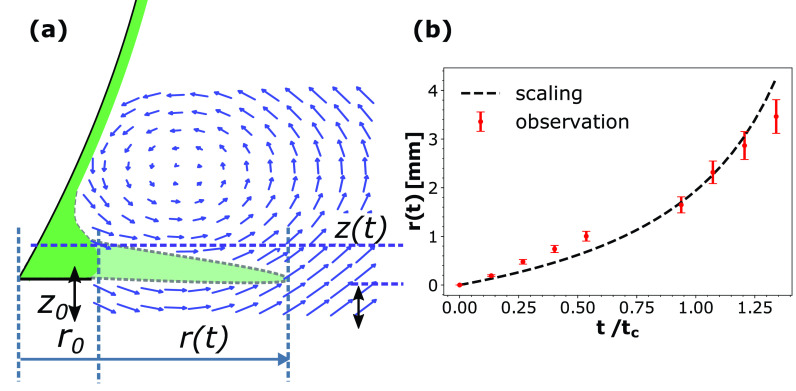
(a) Schematic representation of local velocity field created by the approaching vortex and the initial sheet expansion. r(t) and *z*(*t*) denote the instantaneous measure of the position and thickness of the expanding sheet, respectively. *r*_0_ and *z*_0_ are the initial radial coordinate and thickness of the tear layer accumulated at the bottom of the eye, respectively. The vector field is a schematic representation of the expanding leading vortex. (b) Tear sheet position r(t) from scaling and experiments during the first 5 ms. Capillary time scale $t_{c}=\rho_{l}^{1/2}R^{3/2}\sigma_{al}{}^{-1/2}$ has been used to normalize time.

In order to understand tear sheet expansion, we revert back to phase B. The local velocity field outside the tear film becomes larger and larger as the vortex approaches the cornea. A very thin hydrodynamic boundary layer develops on top of the surface of the tear film, and the boundary layer thickness scales as $\sqrt{\nu t}$ where $\nu =8.91\times 10^{-7}\;m^{2}/s$ is the kinematic viscosity of air at room temperature. During the first 2 ms, the boundary layer thickness approximately becomes 0.04 mm. The viscous effects can be neglected beyond this layer and the sheet expansion is modeled using conservation of mass of the tear film; Fig. 6(a) shows the schematic of the sheet expansion. The dark green layer represents an initial human tear profile. The tear film ejects as a sheet due to the sudden expansion of the approaching leading vortex. Using conservation of mass^47^ of the tear layer accumulated at the base of the eye, a scaling law can be derived for instantaneous position of the envelope of the expanding sheet

$$\rho_{l}zrw=m(t),$$ (4)

where *z* is the thickness of the initial tear film liquid sheet, *r* is the position of the envelope of the tear film sheet, *w* is the width of the tear film, and *m*(*t*) is the mass of the expanding tear sheet. For the initial part of the expansion, r,  z≪w and the changes in *w* is almost negligible compared to changes in *r* and *z*. Therefore, Eq. (4) can be rewritten as

rz=km(t), (5)

where $k=1/\rho_{l}w$. Differentiating Eq. (5) with respect to time (assuming the sheet does not undergo breakup into droplets during the initial phase of sheet expansion), we have

$$\dot{z}r+\dot{r}z=0,$$ (6)

$$\dot{z}r=-\dot{r}z.$$ (7)

Assuming an approximately constant scale of air velocity *V_air_* we have, $\dot{z}∼-V_{\mathrm{air}}$ and thus $z∼z_{0}-V_{\mathrm{air}}t$, where *z*_0_ is the initial thickness of the tear film accumulated at the bottom of the eye. Therefore, Eq. (7) becomes

$$\dot{r}∼\frac{V_{\mathrm{air}}}{z_{0}-V_{\mathrm{air}}t}r.$$ (8)

Integrating and separating the variables in Eq. (8), we have

$$\int \frac{dr}{r}∼\int \frac{V_{\mathrm{air}}dt}{z_{0}(1-V_{\mathrm{air}}t/z_{0})}.$$ (9)

Solving Eq. (9), the scaling form of *r*(*t*) can be written as

$$r(t)=\frac{A}{1-t/B},$$ (10)

where *A* and *B* are constants related to scaling proportionality constant, liquid density, air velocity, and initial conditions. Figure 6(b) shows the scaling form of *r*(*t*) as given by Eq. (10) and the experimental observation of the sheet position (*r*). The maximum standard deviation in the experimental data was about seven percent for 45 sets of temporal data. This deviation occurs due to minute variations in the amount of artificial tear added as well as the natural shape variation in the human eye among difference subjects.

**FIG. 7.**
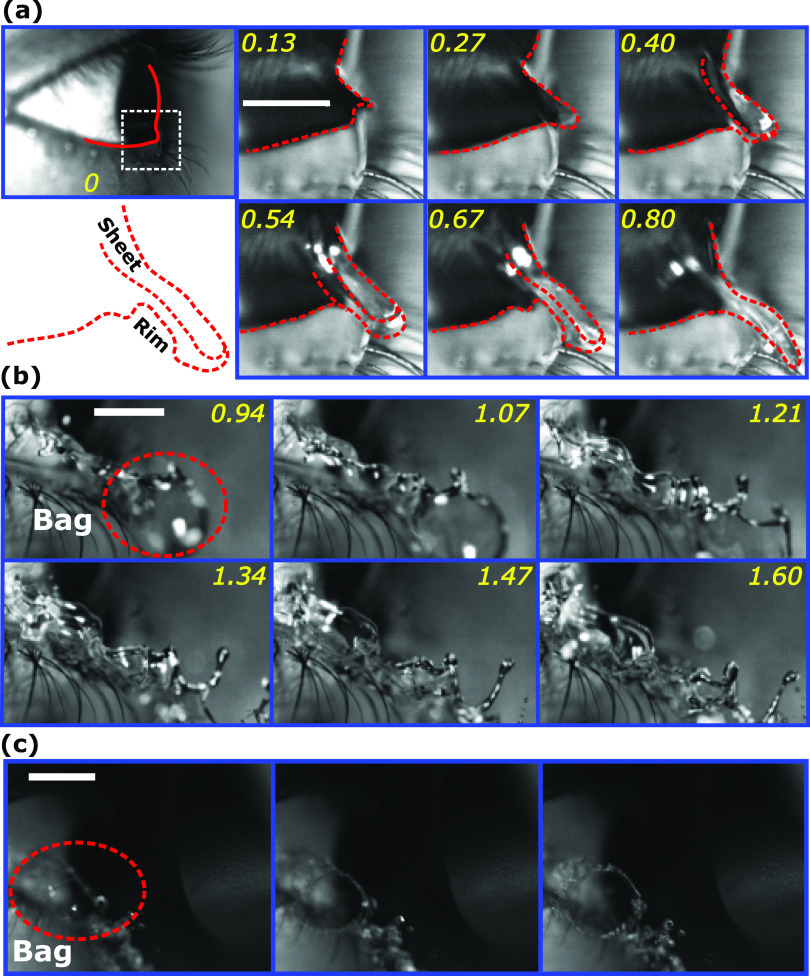
Capillary time scale $t_{c}=\rho_{l}^{1/2}R^{3/2}\sigma_{al}{}^{-1/2}$ has been used to normalize time. The time stamps are non-dimensional. (a) Image sequence shows initial sheet expansion under vortex shearing. (b) Aggravated sheet expansion due to jet impingement at a later stage. Scale bar represents 3 mm and the snapshots are 0.5 ms apart. The red dotted ellipse shows bag like structures (refer multimedia view of Fig. 1) formed in expanding liquid sheets. (c) Head-on view of bag rupture during later sheet expansion. Scale bar represents 6 mm. The rim attached with a bag can been as a circular structure that disintegrates into droplets by Rayleigh–Plateau instability.

Figure 6(b) shows two stages of expansion. The initial expansion of the sheet under vortex expansion and shearing (linear region upto 2 ms) is further aggravated by the kinetic energy of the impinging jet (after 2 ms). The sheet expansion in the second stage is more complicated due to three-dimensional effects. As the sheet expands beyond the eye, it appears to be bounded by a rim [Fig. 7(a)] due to the liquid–air interfacial tension. The sudden sheet expansion also causes small disturbances to appear. These disturbances occur in the azimuthal direction (along the curved perimeter of the growing rim of the expanding sheet) relative to a cylindrical coordinate system attached with the expanding sheet (center of eye is the origin). Further expansion leads to the onset of Rayleigh–Taylor instability,^48^ which amplifies small perturbations into finger like structures as shown in Fig. 7(b). The azimuthal symmetry is broken due to the occurrence of transient structures. However, the local symmetry, such as the distance between the fingers (λ) is preserved, which is useful for the analytical discussion presented in Sec. III E 2.

#### 2. Linear stability analysis of the rim

**FIG. 8.**
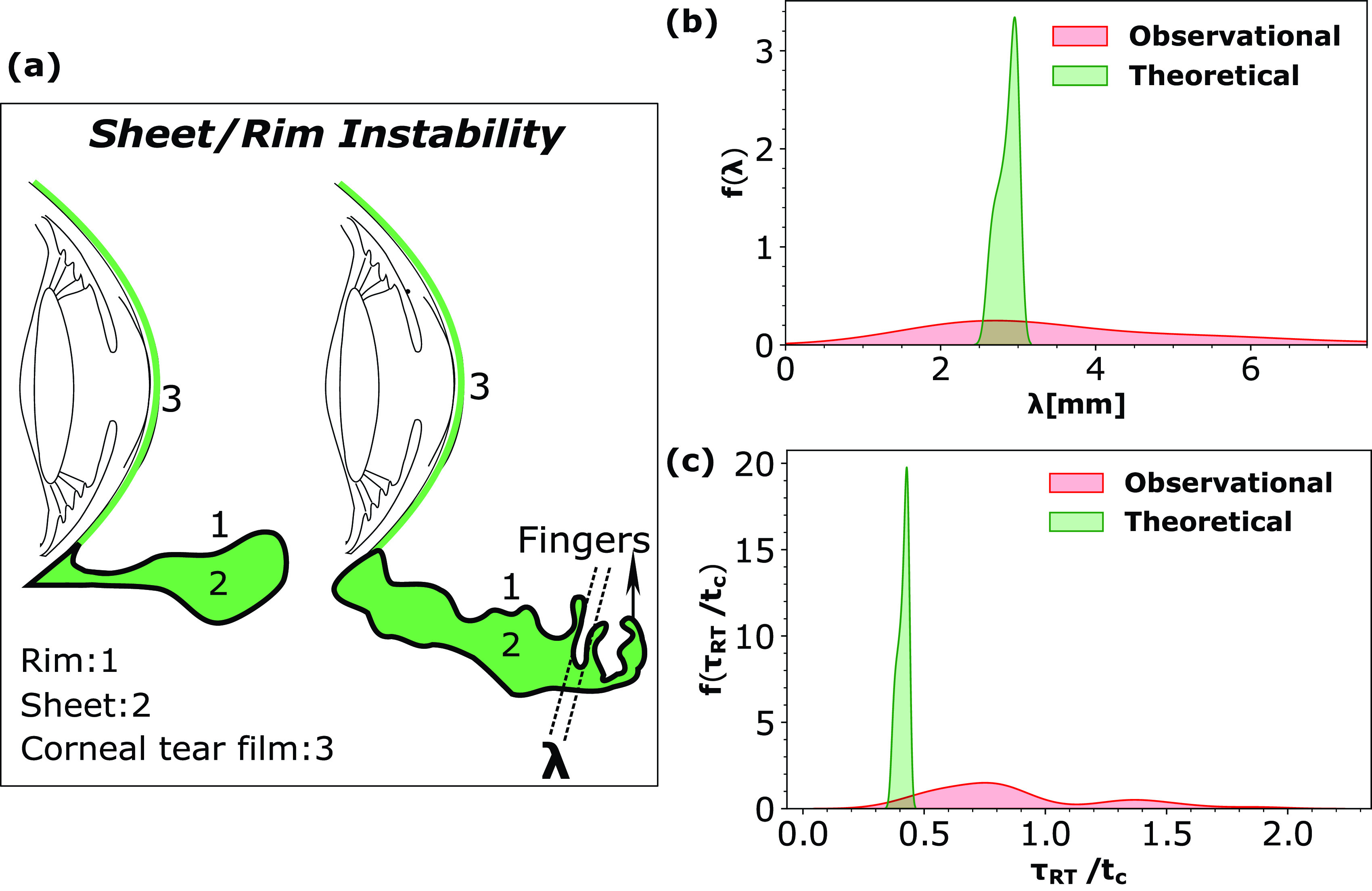
(a) Schematic representation of sheet/rim instability leading forming finger like structures separated at a distance of λ. (b) Probability density function for inter-finger distance λ (theoretical and experimental). (c) The probability density function for the finger formation time scale $\tau_{RT}$ (ms) (theoretical and experimental). Probability density functions were calculated using kernel density estimate technique utilizing the Gaussian kernel. Capillary time scale $t_{c}=\rho_{l}^{1/2}R^{3/2}\sigma_{al}{}^{-1/2}$ has been used to normalize time.

The high initial acceleration of the sheet ($g_{\mathrm{eff}}∼350\;{m/s}^{2}$) during the short time (3.5 ms) allows initial perturbations (in the velocity and pressure field) in the expanding rim of the sheet to develop into finger like structures as shown in Fig. 8(a). We used one-dimensional (1D), linear stability analysis in the azimuthal direction (along the perimeter of the expanding rim)to estimate the length scale (λ) of the growing fingers [see Fig. 8(a)] and the associated finger formation time scale represented as $\tau_{RT}$. The general Rayleigh–Taylor dispersion^31,32,48^ relation for the 1D case is given by

$$\omega =\frac{1}{2}i\left(\frac{\rho_{l}-\rho_{a}}{\rho_{l}+\rho_{a}}\right)kV\pm {\left(I+II-\mathrm{III}\right)}^{1/2},$$ (11)

where *V* is the relative velocity between the expanding sheet and the surrounding air and the terms *I*, *II*, and *III* are expressed as follows:

$$I=\frac{\rho_{a}\rho_{l}}{{\left(\rho_{a}+\rho_{l}\right)}^{2}}k^{2}V^{2},$$ (12)

$$II=\frac{\left(\rho_{l}-\rho_{a}\right)}{\left(\rho_{l}+\rho_{a}\right)}g_{\mathrm{eff}}k,$$ (13)

$$\mathrm{III}=\frac{\sigma_{al}}{\left(\rho_{l}+\rho_{a}\right)}k^{3},$$ (14)

where *g_eff_* is the apparent initial acceleration of the expanding sheet [left eye; Fig. 7(a)] and k=2π/λ is the wavenumber. The real part of Eq. (11) has contributions from three terms: *I*, *II*, *III*. The first term *I* signifies the coupling of one fluid's kinetic energy with respect to the other and its effects on the angular frequency of the perturbations. The second term *II* signifies the apparent initial acceleration felt by the expanding sheet relative to the surrounding air. The third term *III* is the contribution due to the liquid–air surface tension of the sheet. A linear stability analysis on Eq. (11) is performed. The value of *k* where Re(ω) (real part of ω) is maximum, i.e., the wavenumber of the fastest growing perturbations is given by the solution of the following equation:

$$k^{2}-Bk-C=0,$$ (15)

where $B=\frac{2\rho_{a}\rho_{l}V^{2}}{3(\rho_{a}+\rho_{l})\sigma_{al}}$ and $C=\frac{(\rho_{l}-\rho_{a})g_{\mathrm{eff}}}{3\sigma_{al}}$

$$k_{\text{max}}=\frac{2\pi }{\lambda }=\frac{B}{2}+\sqrt{\frac{B^{2}}{4}+C}.$$ (16)

The solution of Eq. (15) is the wavenumber ($k_{\text{max}}$) given by Eq. (16). The other solution for *k_max_* of the quadratic equation (15) is disregarded as it is trivially negative. Figure S1 in the supplementary material (refer Fig. S1 here) shows the Rayleigh–Taylor dispersion profiles for three different relative velocities of the airfield with respect to the sheet (1, 4, and 8 m/s). Figure 8(b) plots the probability density functions of the physically observed λ as well as theoretical estimation of λ calculated using Eq. (16). The experimental probability density function for the distance between the fingers (λ) is obtained from 42 data points. The density function peaks around λ∼2.5 mm and has a standard deviation of λ ∼1.7 mm. A good match is observed between the peaks of the theoretical and experimental distributions corroborating the theoretical and experimental scales. Figure 8(c) shows a similar plot of probability density function for the time scale $\tau_{RT}$. The time scale $\tau_{RT}$ is proportional to the reciprocal of Re(ω). The finger formation time scale ($\tau_{RT}$) experimental probability density function is obtained from 38 data points. The density function peaks around $\tau_{RT}∼2.75\;\text{ms}$ and has a standard deviation of $\tau_{RT}∼1.3\;\text{ms}$. Observed time scale of finger formation is of the same order of magnitude as the predicted scales of $\tau_{RT}$, validating the scaling analysis. The deviation between experiments and theory is due to the multi-modal nature of the probability density functions. The multi-modal nature is attributed to the [from Eqs. (11) and (16)] relative velocity of the tear sheet with respect to surrounding airflow (*V*) and associated acceleration (*g_eff_*). The theoretical probability density functions are plotted for a range of *V* but for a single value of *g_eff_*. The *g_eff_* corresponds to the most probable value observed from experiments.

### F. Disintegration of fingers into droplets (phase F)

**FIG. 9.**
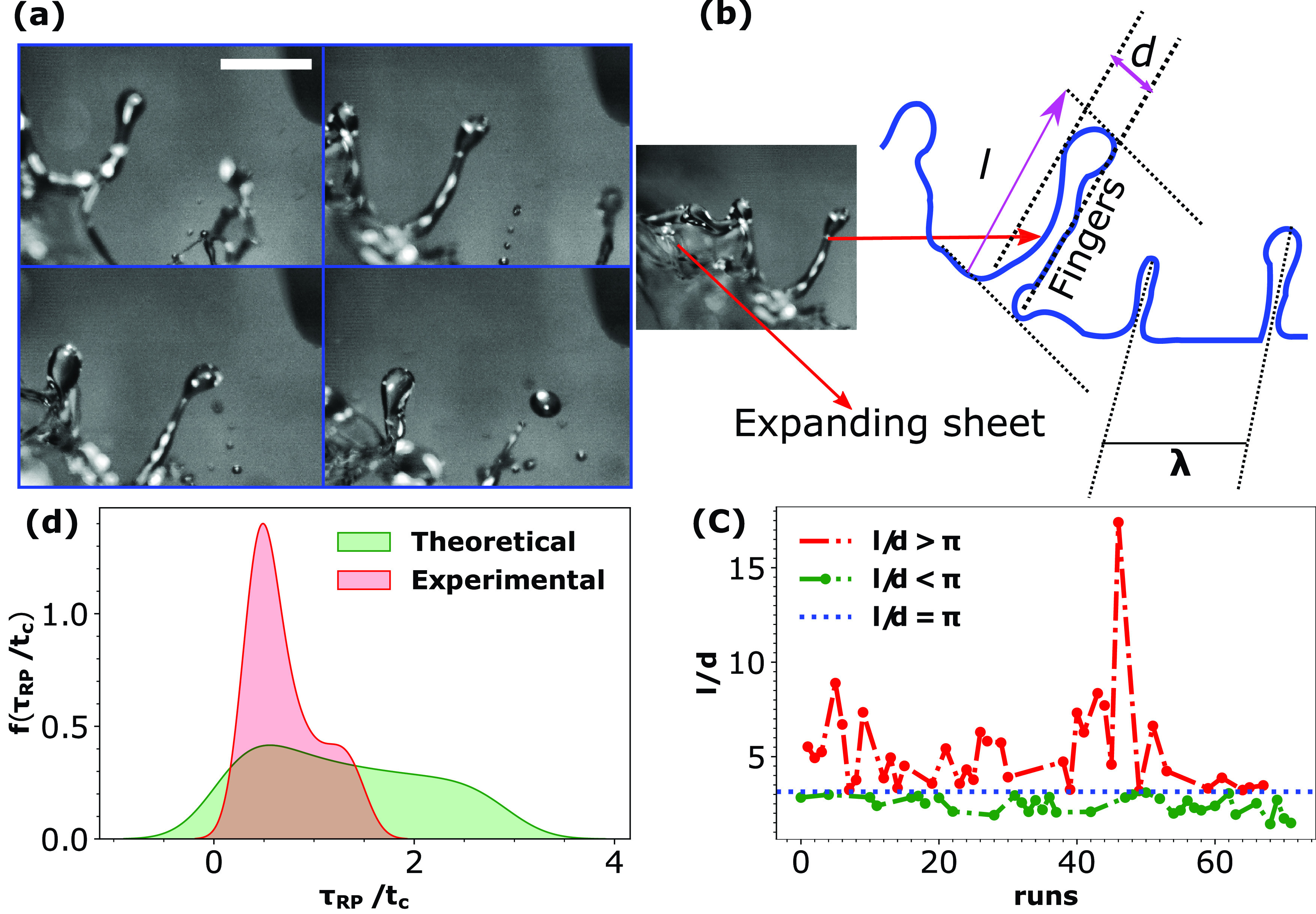
(a) Sequence of images showing the Rayleigh–Plateau breakup. Time stamps are in milliseconds. Scale bar equals 3 mm. (b) Schematic representation of finger like structures showing the maximum length (*l*), width (*w*), and inter-finger distance (λ). (c) The ligament aspect ratio (*l*/*d*) plotted for 60 different fingers to corroborate the Rayleigh–Plateau breakup criterion. (d) The probability density function for ligament breakup time scale $\tau_{RP}$ (ms) calculated using density estimate technique incorporating the Gaussian kernel. Capillary time scale $t_{c}=\rho_{l}^{1/2}R^{3/2}\sigma_{al}{}^{-1/2}$ has been used to normalize time.

A transient phenomenon observed during sheet expansion is the formation of bag like structures [refer multimedia view of Figs. 1, 7(b), and 7(c)]. This is due to the highly transient and three-dimensional nature of the relative airflow with respect to the expanding sheet [Fig. 7(b)].^28^ The Weber number relative to the gas phase is approximately 2 and relative to the liquid phase is approximately 80.^29^ Jaberi and Tadjfar showed that bag like structures [Figs. 7(b) and 7(c)] can be formed in expanding sheets under the influence of a surrounding flow field. The Weber number reported in their work is consistent with the observations of the expanding tear film liquid sheet. These along with the finger/ligament like structures formed by the Rayleigh–Taylor mechanism undergo ligament breakup at the droplets^48^ to create droplet projectiles as shown in Fig. 9(a).

#### 1. Rayleigh–Plateau analysis of droplet formation

**FIG. 10.**
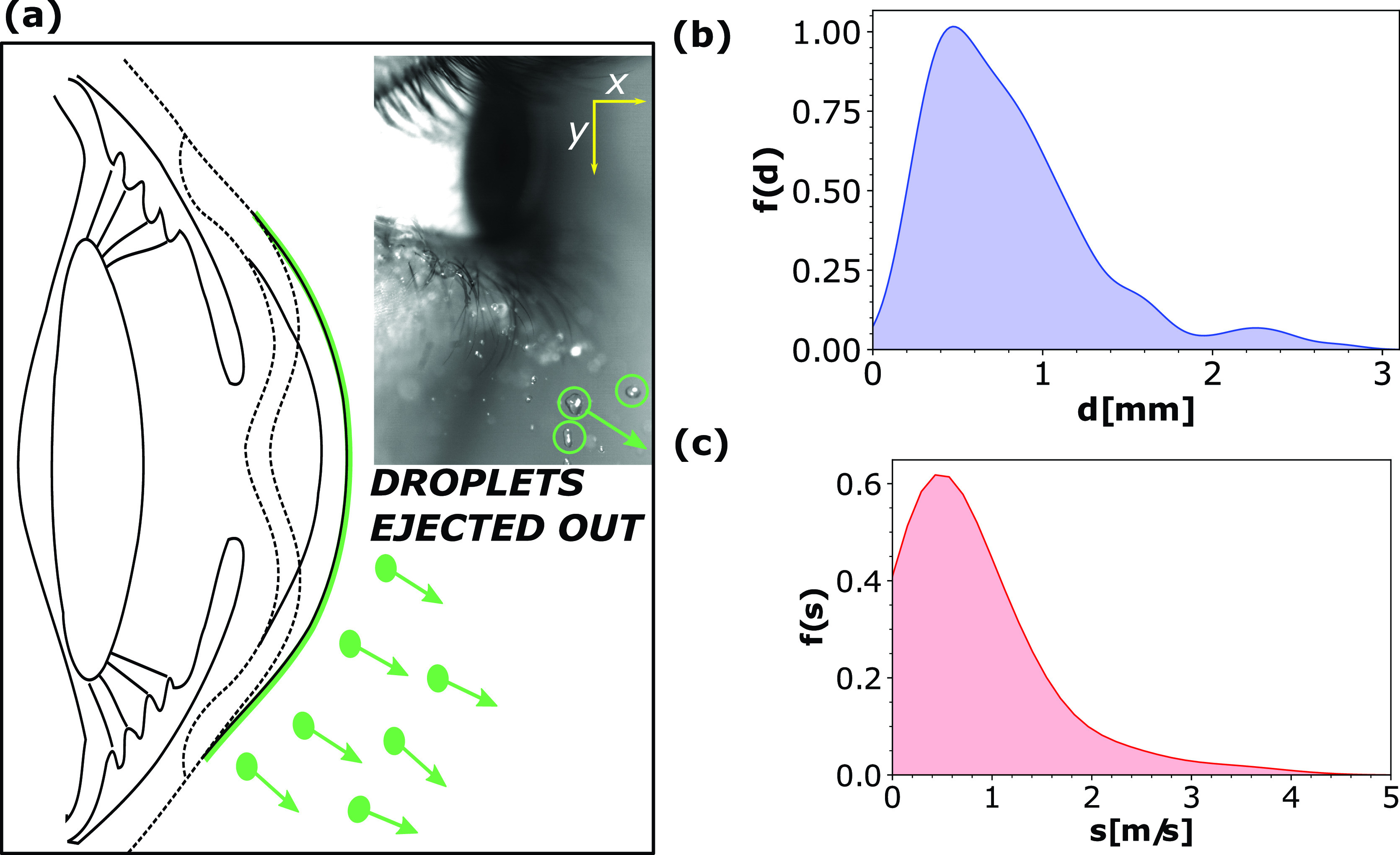
(a) Schematic representation of droplets ejected from a watery eye. (b) The probability density function for droplet size *d* in mm. (c) The probability density function for ejected droplets' speed *s* in m/s. The density functions were calculated using Gaussian kernel.

The dispersion relation for 1D Rayleigh–Plateau stability analysis is given by^31,32,48^

$$\omega^{2}=\frac{\sigma_{al}}{\rho_{w}t_{0}^{3}}kt_{0}\frac{I_{1}(kt_{0})}{I_{0}(kt_{0})}(1-k^{2}t_{0}^{2}),$$ (17)

where *I*_0_ is the modified Bessel function of the first kind (zeroth order), *I*_1_ is the modified Bessel function of the first kind (first order), *t*_0_ is the ligament width, and *k* is the wavenumber. The maximum width and the length of the ligament prior to retraction/tip breakup are $t_{0}=l$ and *d*, respectively. Only ligaments with aspect ratio l/d>π^48^ extend and break into droplets while those with smaller aspect ratios (l/d<π) stay intact. We experimentally validated the breakup criterion for 60 fingers as shown in Fig. 9(b). The red dashed curve corresponds to ligaments that disintegrated into droplets while the green curve corresponds to ligaments that simply retracted after extending. The Rayleigh–Plateau tip break time scales as $\tau_{RP}∼1/\omega_{\text{max}}$ and is associated with the angular frequency corresponding to the fastest growing perturbation, causing the ligament pinch-off into droplets. The theoretical dispersion curve (refer Fig. S2 here) was calculated using Eq. (22) from the Rayleigh–Plateau linear stability theory. The value of ligament thickness ($t_{0}$) is assumed to be of the same order of magnitude as the rim. Observed values of the rim thickness were widely distributed. We selected two extreme values of $t_{0}$: 0.22 and 0.43 mm. As shown in Fig. S2, thicker ligaments have faster growth rates ($\tau_{RP}=1/\omega$) but have the same value of $kt_{0}∼0.71$. Figure 9(d) shows the experimental and theoretical probability distribution functions for the ligament breakup time. The ligament breakup time scale ($\tau_{RP}$) experimental probability density function is obtained from 56 data points. The experimental distribution peaks at $\tau_{RP}∼1.6\;\text{ms}$ and has a standard deviation of $\tau_{RP}∼1.3\;\text{ms}$. The theoretical and the experimental curves peak at the same value, thus corroborating Rayleigh–Plateau linear stability analysis. The droplets from Rayleigh–Plateau breakup [depicted in Fig. 10(a)] exhibit wide distribution in size and velocity. The coordinate system [inset Fig. 10(a)] used to calculate the velocity of the droplets is elaborated in the supplementary material (refer Fig. S3 here). A bounding rectangle was used to calculate the shape of the droplet. The droplets' major axis *a* and minor axis *b* were calculated from the bounding rectangle's width and height, respectively. Mostly, the droplets were ellipsoidal in shape. The projected droplet diameter could be expressed as $d={\left(ab\right)}^{1/2}$ and varies between 0.2 and 3 mm. Figure 10(b) plots the probability density function for the projected diameter *d* in mm. The most probable droplet size is approximately 0.4 mm as it can be seen from the peak in Fig. 10(b). The individual droplet velocity was calculated from the derivative of its position using a forward difference scheme. The probability density function for the speed (*s* in m/s) of the ejected droplets is shown in Fig. 10(c). The droplets ejected have a wide spectrum of speed from 0.1 to 5 m/s. The most probable velocity however peaks at approximately 0.2 m/s.

## IV. CONCLUSION

The tonometer ejects an air puff that is essentially a leading vortex trailed by a jet. The vortex ejected at 4–5 m/s slows down on reaching the eye. Its kinetic energy creates a transient pressure reduction, forcing the corneal tear film (watery eyes) to expand beyond the eye. Shortly after the initial expansion commences, the trailing jet impacts the cornea, propagating capillary waves, which accelerate the sheet leading to rim instability. Due to the rapid rate of expansion, small perturbations at the sheet's expanding rim transform into finger like structures due to Rayleigh–Taylor instability. Due to the highly transient and three-dimensional nature of the flow field, bag like structures also appear and disintegrate into ligaments. Finally, both the fingers from Rayleigh–Taylor as well as the ligaments from the bag formation undergo Rayleigh–Plateau instability and disintegrate into ellipsoidal droplets measuring 0.2–3 mm. In a previous study,^14^ we measured the mean airflow in a standard hospital setting as 0.5–1 m/s. The maximum spread radius^20^ of the smallest droplets (0.2 mm in size) generated during tonometry is ∼0.5 m, which is larger than the distance between the patient and the tonometer (11 mm). Thus, there is a high probability that patients with watery eyes (naturally occurring due to chronic eye conditions or artificially administered lubricants) can generate potential carriers of pathogens. These may deposit on the surface of instruments like the tonometers, converting them to fomites and in the absence of adequate sanitization process, transmit viruses like SARS-CoV-2.

In conclusion, we present a detailed mechanistic understanding of how energetic gas flows can atomize a thin liquid film into droplets. We have used linear stability theory to predict the length scales and the time scales involved in the journey of the tear from sheet to droplets. These were also corroborated by high-speed imagery. In the context of medical tests such as the tonometry, it is apparent that routine procedures can lead to droplets forming aerosols as well as fomites. We would also like to re-iterate that due to experimental limitations, the tear film breakup could contain a smaller size of droplets. Potentially, these droplets could be transmitted beyond 0.5 m although their infectivity could be limited by evaporation (see Basu *et al.*^22^).

## V. MATERIALS AND METHODS

The schematic of the experimental setup is shown in Fig. S4 of the supplementary material (refer Fig. S4 here). The experiments were conducted on four different human subjects of various age groups (in the range of 24–32) and gender (one female and three males) at room temperature of 25 °C±1 °C. All the test subjects were COVID negative and had given proper consent before volunteering as test subjects. Images were acquired using a high-speed camera (Photron Mini UX100) and a high-intensity light source (Veritas Mini Constellation) at 2000–10 000 frames per second with a spatial resolution of 512 × 320 pixels. Shin-Nippon NCT 200 (Rexxam Co. Ltd., Osaka, Japan) was used as the non-contact tonometer for all the experiments. High fidelity images were captured from the side, orthographic, oblique, inclined, and back views to obtain detailed kinematic information of the highly transient and three-dimensional phenomena. The high-intensity light source was placed in different strategic locations to attain the desired lighting. Fluorescence imaging was also used to visualize the droplets generated from the watery eye. Further details of the experimental setup and fluorescence imaging can be found in our previous work.^14^

The tonometer's nozzle was kept at a working distance of approximately 11 mm from the human eye following the standard protocol of the NCT200 instrument for a correct reading of intraocular pressure, and the nozzle height was 0.5 m from the table top. The initial vortex and trailing jet were observed by smoke flow visualization and scattering techniques.^49^ Human subjects were replaced by a cadaver goat eye for smoke flow visualization to characterize the air puff coming out of the tonometer and understand the leading vortex and the trailing jet's roles. The cadaver eye was kept at a working distance of approximately 8 mm from the tonometer nozzle exit. Experiments were conducted on two different eye conditions: dry and wet. Dry eye conditions were studied to have a comprehensive understanding of the corneal deflection and observe whether any droplets are ejected. The wet eye conditions were simulated using eye drops that were prescribed by an ophthalmologist. Some industrial brands of eye drops used are Refresh Tears, Lubrex, Trehalube, and Systrane Ultra. The drops were used on the human subjects just before the tonometry measurement to mimic watery eye conditions. The images were acquired with Photron FastCam Viewer (software package 4.0.3.4.), and the raw images were processed using a combination of open-source tools like ImageJ^50^ and python.^51^ About 125 experimental runs were conducted on all four subjects to ensure statistically significant datasets. Preprocessing of the raw images was performed using FFT bandpass filter^52^ and CLAHE (contrast limited adaptive histogram equalization).^53^ The significant structures were filtered down to 40 pixels, and small structures up to 3 pixels were used for the FFT bandpass filter. For CLAHE, a block size of 127 pixels was used along with histogram bins of 256 and a maximum slope of 3. The various kinematic parameters involved were then tracked, and the results were post-processed using Python.^51^ The discrete raw data of the kinematic parameters were converted into a continuous probability density function using kernel density estimate. The Gaussian kernel has been used for the kernel density estimate. For simplistic purposes, the tear film properties are taken to be the same as those of liquid water, which does not change the underlying mathematics, scaling laws, and the related fluid dynamics of the different processes involved at the relevant time scales under consideration.

## SUPPLEMENTARY MATERIAL

See the supplementary material for Raylor–Taylor dispersion curves for different airflow velocities (V) with respect to the expanding sheet. The three cases shown are for V = 1, 4, and 8 m/s, respectively (Fig. S1); Rayleigh–Plateau dispersion curves for different ligament (0.43, 0.22 mm) thicknesses (Fig. S2); coordinate system used for calculating various geometric and kinematic parameters like droplet size and velocity (Fig. S3); schematic of experimental setup: (1)—non-contact tonometer, (2)—high-speed camera, (3)—light source, (4)—computer, (5)—tonometer operator, (6)—human subject (Fig. S4).
